# “Mind the Gap”—Differences between Documentation and Reality on Intensive Care Units: A Quantitative Observational Study

**DOI:** 10.3390/healthcare12151481

**Published:** 2024-07-26

**Authors:** Florian Jürgen Raimann, Cornelius Johannes König, Vanessa Neef, Armin Niklas Flinspach

**Affiliations:** Department of Anesthesiology, Intensive Care Medicine and Pain Therapy, University Hospital Frankfurt, Goethe University Frankfurt, 60590 Frankfurt, Germany

**Keywords:** critical care, catecholamines, patient safety, risk management, sedation

## Abstract

Introduction: Digitalization in medicine is steadily increasing. Complex treatments, scarce personnel resources and a high level of documentation are a constant burden on healthcare systems. The balancing between correct manual documentation in the digital records and limited staff resources is rarely successful. The aim of this study is to evaluate the adherence between documentation and lack of documentation in the treatment of critical care patients. Material and Methods: For the evaluation, data from the hospital information system (HIS) of several intensive care units (ICU) were examined in conjunction with data collected from a checklist. All boluses of sedatives, analgesics and catecholamines were documented paper based across all shifts and all weekdays and compared with corresponding digital data from the HIS (2019–2022) of previous years. Results: 939 complete digital patient records revealed a massive under-documentation of the medication administration compared to that applied according to the checklist. Only 12% of all administered catecholamines, 11% of α_2_-agonists, 33% of propofol, 92% of midazolam and 46% of opioids were found in the digital recordings. In comparison, the effect was more pronounced on weekdays compared to weekends. In addition, the highest documentation gap was found in the comparison of early shifts. Comparing neurosurgical vs. internal vs. anesthesiologic ICUs there was a highly significant difference between anesthesiologic ICUs compared with other disciplines (*p* < 0.0001). Discussion: Our data shows that there is a remarkable documentation gap and incongruence in the area of applied boli. Automated documentation by connecting syringe pumps that enter data directly into the HIS can not only reduce the workload, but also lead to comprehensive and legally required documentation of all administered medication.

## 1. Introduction

Modern intensive care medicine has undergone extreme change since its emergence in the mid-50s of the last century [1]. While initially focusing on patient monitoring and rapid recognition of critical situations for required interventions, the situation has evolved considerably. With mechanical ventilation, continuous application of pharmaceuticals and organ replacement techniques, the complexity and costs increased drastically [2]. In order to be able to ensure the justification of these costs, regulatory authorities and billing auditors have also increased considerably. Even though digitalization and networking have already reduced the manual input of data in many aspects, the majority of data is still generated via manual input. The use of digital vital parameter monitoring and automatized data feeds of devices such as ventilators reduced the amount of manual data entry; however, the technical evolution of the last decades has resulted in increasingly detailed and considerably more complex care with consecutive documentation. Examples include early physiotherapeutic mobilization, positioning therapy to prevent decubitus and contractures and patient-adapted nutritional therapy as relevant labor-intensive steps. For this reason, many countries have a staffing threshold to ensure sufficient allocation of personnel resources for such complex cases. The documentation of medical findings, as well as the nursing and general care measures taken, plays an important role in ensuring the success of treatment as well as a forensic role [3]. Digital documentation of these findings has a number of advantages, particularly in terms of retrievability and secure storage, and an ongoing process of improvement has been underway for many years [4].

Providing sufficient evidence of correct treatment requires increasingly high-quality and precise nurses and physicians [5]. In particular, the administration of pharmaceuticals and possible interactions between these substances are increasingly important, especially in the collective of pre-existing polypharmacologically treated, multimorbid patients. Unfortunately, there is an increasing lack of qualified staff in intensive care units, which leads to an increase in workload [6]. This dichotomy between the increasing requirements of documentation on the one hand and the challenging circumstances in staffing of the 21th century and complexity in patient treatment on the other constitutes a problematic issue. In particular, the manual documentation of individual, but certainly therapy-relevant, medication boluses being correctly documented in the patient files may be questioned. It is precisely this manual documentation of boluses of continuously administered medications that appears vulnerable to being lost under the existing workload. In the Federal Republic of Germany, the obligation to provide complete documentation of patient treatment lies upon the practitioner. If the documentation is incomplete, the burden of proof is reversed, so that the evidence of an incorrect procedure is transferred from the patient’s burden of proof due to inadequate documentation to the practitioner, who must then prove that the error complained of was not caused by the treatment provided. The treating clinic or the primary treating specialist is considered to be the practitioner in a strict sense, although according to the legal situation, the duty of documentation can be delegated to qualified staff such as junior physicians and nursing staff in the case of nursing activities. A similar legal procedure with regard to the burden of proof based on the medical or delegated treatment documentation can also be found in numerous other European countries (France, Austria, Switzerland) [7,8,9,10]. This means that in the case of forensically relevant treatment documentation, the precise and careful recording of all relevant treatment steps must be carried out by the physicians responsible for documentation and, in the case of bolus applications, by intensive care nurses. Any resulting legal disputes can have consequences ranging from civil law to individual criminal law consequences, depending on the error complained of (persistent physical injury to negligent homicide). The fundamental positivist assumption of conscientious and comprehensive documentation of all treatment procedures carried out on the patient proves to be difficult to maintain, especially in the case of a forensic assessment, even if it is rarely carried out. In terms of adequate risk management, it is precisely this aspect, which is critical to treatment and relevant to claims, that should lead to a regular review irrespective of any specific event. Our study intended to conduct a corresponding independent voluntary review of the practiced documentation compliance in a center of excellence.

In a clinical situation of divided documentation of digitally recorded syringe pump run rates and separate manual entry for bolus documentation, our study project aimed to evaluate any existing data gap.

## 2. Material and Methods

The study was conducted in accordance with the ethical principles of the Declaration of Helsinki (Ethical Principles for Medical Research Involving Human Subjects) [11]. For the collection of the syringe pumps data on which the calculation was based, the study protocol was approved by the ethics committee of the institution (No. 2022-995) and a waiver of written informed consent was approved. Verbal informed consent was obtained for the collection of data from employees. This manuscript complies with current EQUATOR guidelines [12]. The study was conducted at a tertiary university center in Germany. The treatment data evaluated in the study related to the largest intensive care unit of this tertiary center, which covered the care of critically ill patients from various surgical disciplines, including cardiac surgery patients. In addition, care for critically ill COVID-19 patients was provided on the ward during the pandemic.

### 2.1. Data Acquisition

To determine differences between the manually documented bolus applications, a bedside checklist was created. This checklist recorded the regularly used catecholamines (norepinephrine bolus á 10/20/30/40/100 µg and epinephrine bolus á 10/20/30 µg), sedatives (propofol bolus á 10/20/30/40 mg, midazolam bolus á 1/2/3/4/5/8/10 mg and clonidine bolus á 50/100/150 µg) and opioids (sufentanil bolus á 5/10/15 µg) with the possibility of using a tick list to fill in the regularly administered bolus doses. In addition, the checklist for evaluation recorded the respective day of the week and the shift considered during the documentation (early, late, night shift); the original checklist can be found in Supplement I. Likewise, the procedure described at the center for digital documentation based on a separation of responsibilities is both a national standard and a regular international practice. In Germany, documentation obligations are also set out in the professional regulations of the professional groups involved, both medical and nursing. After extensive training, the nursing staff were instructed to enter the basic information (shift type and the day) at the beginning of the shift, as well as any medication currently being administered to the patients under their charge. During the shift, each bolus administered was to be documented by a tick in the checklist in addition to the information in the electronic patient data management system (PDMS; Metavision 5.4, iMDsoft, Tel Aviv, Israel). To obtain a representative sample, 939 questionnaires were collected in the period from 1st November 2023 to 31th January 2024. The checklist results were recorded in Excel (Version 365, Microsoft Corp., Redmond, WA, USA).

#### 2.1.1. Inclusion Criteria 

All beds in the aforementioned intensive care units of the hospital (anesthesiological-surgical intensive care unit, neurosurgical intensive care unit, internal medicine intensive care unit) in which the same PDMS (MetaVision 5.4) was used for documentation. 

#### 2.1.2. Exclusion Criteria

- Wards that work on paper or with the help of other software solutions were excluded from the study.
- Wards designated to treat mainly minors or infants (pediatric intensive care units).

### 2.2. Statistical Analysis

Prior to this study, a calculation was carriedout to ensure a representative sample size of one month of critical care patient treatment in terms of the checklists to be recorded. The sample size is therefore based on the requirement to collect at least 900 completed questionnaires during the study period. The categorical variables are presented as counts and percentages. Variables that are not normally distributed are described as medians (interquartile range IQR [25/75]). Differences between pharmacological groups were assessed using Fisher’s exact test for categorical variables with Welch’s correction. One-way analysis of variance (ANOVA) was used for comparisons between groups and for multiple comparisons in addition to Turkey’s and Sidak’s multiple comparisons test.

All statistical tests were two-sided tests and results with *p* < 0.05 were considered statistically significant. All analyses and graphs were performed with SPSS^®^ (IBM Corp., Version 29, Chicago, IL, USA), GraphPadPrim 7 (Boston, MA, USA).

## 3. Results

**Figure 1:**
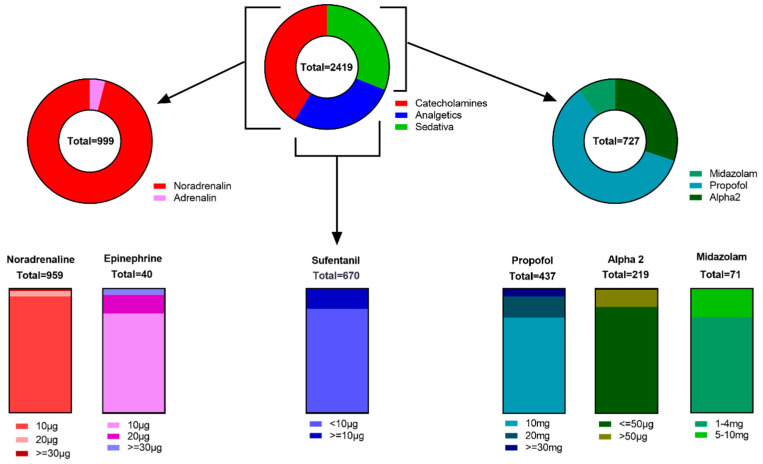
Graphical illustration of the percentage of boluses observed. Graphical illustration of the percentage of boluses observed in the pie chart with sub-differentiation of the three pharmaceutical groups and substances. Abbreviations: µg, microgram; mg, milligram.

During the study period, we were able to assess 939 fully completed documentation forms. These checklists contained 2419 individual boluses of catecholamines (999), sedatives (750) and analgesics (670), corresponding to an average month of documentation. Caregivers reported that they did not treat any patients receiving any of the specified continuously administered medications in 225 checklists. A further 226 checklists referred exclusively to the administration of catecholamines; out of the 999 individual boluses of catecholamines observed in total, 96.0% were for noradrenaline and 4.0% for epinephrine. In 20 cases, only the opioid anesthetic sufentanil was administered continuously. A differentiated breakdown of the percentage distribution found can be found in Figure 1.

In 108 of the analyzed checklists, multiple sedation with two or more continuously administered sedatives was necessary to ensure sufficient treatment compliance. In addition, according to the documentation, in 34 cases, only one sedative medication was administered, including 24 cases with use of an alpha2-agonist for moderate distancing.

**Figure 2:**
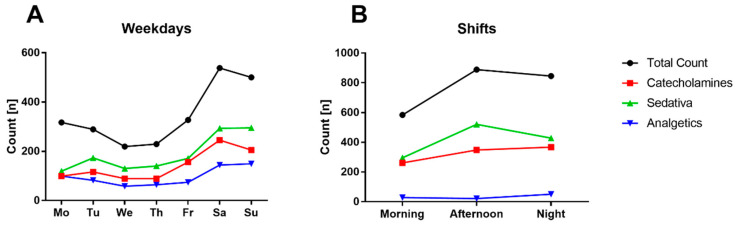
Time breakdown of the boluses found in checklists. Breakdown of bolus distribution in temporal resolution by day of the week (**A**) and within the course of the day of the existing three-shift system (**B**) in relation to the three pharmaceutical groups.

There was also an inhomogeneous distribution of the observed boluses in the checklists, so that a disproportionately high number of boluses were documented on weekend days (Saturday and Sunday, see Figure 2A), as well as a shift-dependent distribution with increased documentation of boluses during night and late shifts (see Figure 2B).

**Figure 3:**
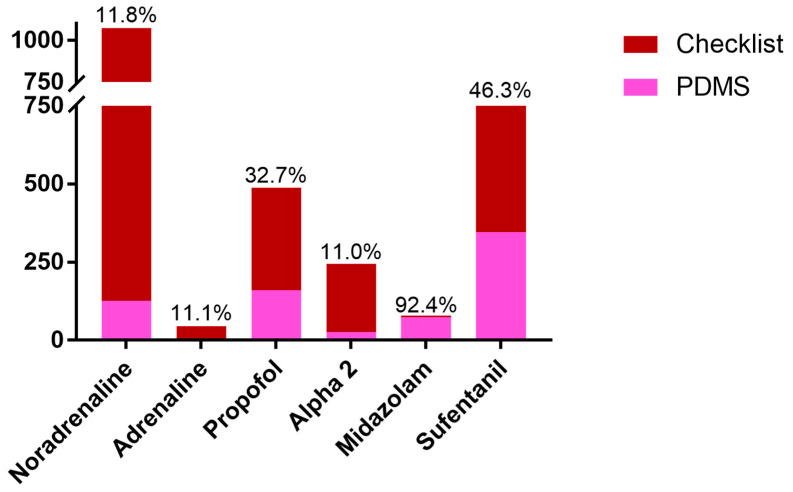
Graphic bars illustrating PMDS data compared to checklist documented boluses. Illustration of the bolus applications taken from the checklist-based sampling with referencing of the boluses recorded in the PDSM (Patient Data Management System) in reference to the pharmaceuticals analyzed. Percentages inserted represent the proportion of boluses recorded in the PDMS system in comparison to the bolus applications documented manually during the survey period.

By comparing the checklist-based recording of bolus applications, taking into account the number of patients, we found that only 12% of the boluses of both noradrenaline and adrenaline were recorded in the digital documentation. With regard to sedatives, 11% of the boluses of α_2_-agonists were found and 33% of the propofol boluses administered were mapped in reference to the existing data set. Regarding the less frequently used midazolam, a reproduction corresponding to 92% documentation was found. The digital documentation found for the opioid sufentanil covered 46% of the checklist-based boluses. A detailed comparison of the checklist-based documentation with the digital documentation can be found in Figure 3.

We also observed that there was a continuous drop in the number of documented boluses over the survey period of 2019–2023.

Based on the above results, a consultation of the in-house pharmacy delivery system revealed a plausible, albeit not quantifiable, gap which underpins the recorded results.

Furthermore, we analyzed bolus applications on the internal and neurosurgical intensive care unit (ICU). Both ICUs operate using the same PDMS, albeit with different staff and a different spectrum of patients. We found that significantly lower numbers of boluses were documented in these ICUs than in the anesthesiology ICU we primarily investigated; a breakdown can be found in Table 1.

## 4. Discussion

The requirements for legally compliant documentation are high and steadily increasing. Forensic reliability concerning recourse claims requires complete documentation, particularly in terms of administered medications [13,14]. According to the nature of intensive medical care for critically ill and unstable patients, it requires frequent bolus applications, e.g., to maintain sufficient circulatory stability. However, these boluses appear to be insufficiently documented on a regular basis [15]. However, only appropriate documentation allows accurate treatment of critically ill patients with comprehensible planning and reviewing of errors leading to an increase of patients safety [16].

For any forensic discourse, it is highly relevant to have an interdisciplinary documentation delegation clarified in advance. Depending on the underlying legal norms in terms of professional regulations and delegable activities, this is partly already specified at national level, otherwise a detailed written directive is required. Ultimately, however, the documentation negligence of individuals remains a global liability risk for the treating and defendant hospital, so that adequate documentation should be observed at an early stage in the interests of adequate risk management.

However, one of the most relevant aspects of the correct documentation of medication boluses is patient safety [17,18]. Unknown medication, especially with the pharmaceutical categories we examined, can quickly have serious consequences. For example, it is conceivable that the application of sedatives or opioid boluses that are not known to the attending physician or the following shift could lead to unnecessary diagnostic imaging with potentially dangerous transports (e.g., cerebral CT scans) in the absence of awakening [19]. This is all the more serious as the correct transfer of information in terms of medication bolus documentation would make it possible to adjust therapy or even antagonize medication (midazolam—flumazenil, sufentanil—naloxone). In the present study, we found a distinct weighting of the documented boluses in the late and night shifts, as well as at weekends. It is difficult to make a clear distinction apart from the compliance of the nursing staff’s voluntary information; it is conceivable that the workplace checks, patient washing and oral care that are regularly carried out during the early shift, in addition to the transfers and preparations for new admissions, could have a negative impact on the correct documentation. One further potential approach of this finding is that patients are regularly admitted to the ICU in the afternoon (admission in the late and/or night shift) following their surgical treatment, for example in cardiac surgery or visceral surgery, stabilized overnight and then discharged from intensive care the next day, usually with good convalescence. Repeated bolus applications to stabilize the patient’s condition are particularly common in this critical phase following surgical treatment, which could explain the corresponding accumulation. Similarly, at weekends there is increased supra-regional healthcare for critically ill emergencies, which could also explain the observed phenomenon. Accordingly, the weekend effect published in different areas could at least partially explain the increase in boluses required [20,21].

The urgent need for complete digitalization of the corresponding documentation can be supported by our data for many reasons. While this would immediately guarantee the consistent medical documentation mentioned above, the corresponding freed-up working time of the nursing staff must also be taken into account. Intensive care nurses spend a relevant part of their available working time documenting boluses administered by syringe pumps. It can therefore be assumed that the working time freed up by automated documentation allows for the optimisation of primary nursing care whilst at the same time increasing staff satisfaction by significantly reducing the burden of documentation.

Although we can only speculate as to the reasons for the decline in documentation over time, there are increasing indications that the enormous overwhelm and frustration caused by a sustained overload among staff has increased massively over the course of the COVID-19 pandemic and that this has led to an emigration from the profession, a refusal to work excessive overtime and also to documentation fatigue [22]. In this context, it appears relevant to note that the workload requirements of manual digitization of the corresponding documentation have already been shown by our group to result in enormous personnel costs outside of primary patient care [23].

The requests we initiated to our pharmacy regarding the quantities of catecholamines supplied in the investigative period revealed that even when estimating potential wastage due to misguided syringe changes, incomplete use and patient transfer to other wards, an underestimation of the bolus applications shown in the checklists can be assumed. This is all the more significant as efforts to provide direct consumption feedback for stock monitoring and order management are being considered as an economization factor in an increasingly digital hospital infrastructure [24].

The investigation we initiated has to be considered with some limitations. Even if it was blinded with regard to the primary outcome, the study is dependent on an honest statement regarding the boluses administered in real life. However, the assumption of correct information is vulnerable due to possible variously motivated incorrect information provided by the caregivers. Another limitation is the small sample size, which is why appropriately scaled follow-up studies are necessary to substantiate the results.

In addition, the digital baseline data was collected during the course of the COVID-19 pandemic. It is conceivable that this could have favored underreporting of applied boli due to the more difficult work with protective equipment.

## 5. Conclusions

This study reveals the considerable difficulties of seamless documentation of medication administration in the event of incomplete digitization procedures. This leads to potential legal problems in terms of the obligation to provide evidence as well as problems with correct treatment planning and patient safety.

## Figures and Tables

**Table 1:** Comparison of boluses within the ICUs.

Medication	Comparison	CI (95%)	*p*-Value
Noradrenaline	anesthesiologic vs. internal ICU 22.0 ± 14.5 vs. 2.0 ± 2.5 *	(0.055)–(0.198)	<0.0001
	anesthesiologic vs. neurosurgical ICU 22.0 ± 14.5 vs. 1.0 ± 1.6 *	(0.066)–(0.209)	<0.0001
Propofol	anesthesiologic vs. internal ICU 27.6 ± 20.6 vs. 1.0 ± 1.7 *	(0.098)–(0.241)	<0.0001
	anesthesiologic vs. neurosurgical ICU 27.6 ± 20.6 vs. 4.2 ± 3.8 *	(0.064)–(0.207)	<0.0001
Sufentanil	anesthesiologic vs. internal ICU 59.5 ± 43.1 vs. 1.3 ± 2.1 *	(0.320)–(0.463)	<0.0001
	anesthesiologic vs. neurosurgical ICU 59.5 ± 43.1 vs. 10.1 ± 8.6 *	(0.230)–(0.373)	<0.0001
	internal vs. neurosurgical ICU 1.3 ± 2.1 vs. 10.1 ± 8.6 *	(−0.161)–(−0.018)	0.0033
Overall	anesthesiologic vs. internal ICU 36.4 ± 33.2 vs. 1.3 ± 2.2 *	(0.087)–(0.156)	<0.0001
	anesthesiologic vs. neurosurgical ICU 36.4 ± 33.2 vs. 5.1 ± 6.7 *	(0.063)–(0.132)	<0.0001
	internal vs. neurosurgical ICU 1.3 ± 2.2 vs. 5.1 ± 6.7 *	(−0.059)–(0.010)	0.252

Comparison of boluses within the ICUs. Based on boluses/patient for the study period of 2019–2022. * Specification of the determined mean values of the documented boluses per calendar month with standard deviation of the corresponding ICU.

## Data Availability

The data cannot be shared publicly. The datasets generated and/or analyzed during the current study are not publicly available due to national data protection laws but are available upon reasonable request from the corresponding author or via the data protection officer of the University Hospital of Frankfurt (datenschutz@kgu.de).
